# Supplementary material for: Are carbon emissions trading and green financial instruments synergistic? -Comprehensive quantitative research based on content analysis
Source: PLoS One. 2024 Mar 7;19(3):e0298601. doi: 10.1371/journal.pone.0298601 (PMC10919601; doi:10.1371/journal.pone.0298601)
Supplement: S1 Data — (ZIP) [file pone.0298601.s001.zip › Data/地级市Z_F工作报告.zip!/║┌┴·╜¡/╥┴┤║╩╨/└┤╫╘╣1⁄2╓┌║┼í╛┬φ┐╦╩2╛▌═°í┐_2015.docx]

各位代表：

现在，我代表市政府向大会报告工作，请予审议，并请市政协委员和其他列席会议的同志提出意见。

一、2014年工作回顾

过去的一年，在市委的正确领导下，在市人大、市政协的有力监督支持下，全市上下牢牢把握稳中求进工作总基调，积极应对经济下行带来的重重压力，努力克服森林停伐引发的系列矛盾，稳步推动改革开放，持续推进转型发展，各项工作取得了新进展。

——扎实推进产业项目建设，新的增长点不断涌现。全年新建续建产业项目61个，完成投资45.6亿元。鹿鸣钼矿正式投产，华能热电主体完工，汇源、佳龙等重大项目快速推进，永旺、黑尊、中盟等产业化龙头项目陆续建成，水伊方、桃山玉温泉等新的旅游项目投入运营。“伊春寒地森林猪”、“友好蓝莓”获批地理标志证明商标。铁力、翠峦等园区共完成投资20亿元，入驻企业152户。与金玛集团共同发起成立了“东北绿色食品产业联盟”，产业竞争力得到进一步提升。

——全面落实停伐各项政策措施，生态文明建设取得新进展。深入实施《大小兴安岭林区生态保护与经济转型规划》和天保二期工程，出台了湿地保护办法等一系列法规制度。碧水中华秋沙鸭保护区通过国家评审。实现了连续11年无重特大森林火灾。积极推动建立“伊春国家公园”，并作为我省唯一申报试点上报国家。全面落实防污减排责任，14个污水处理、5个脱硫脱硝项目建成投用。被确定为全国首批生态文明先行示范区和创建新能源示范市。

——重大活动接力推进，城市功能和形象显著提升。第十三届省运会务实节俭、隆重热烈，实现了“办一届最成功省运会”的目标。第三届森博会共有11个国家26个省份参展，会展经济水平得到提升。“创文”工作持续攻坚，市民文明意识得到增强。在重大活动的牵动下，城市建设提质提速，汇源国际会展中心及八大体育场馆建成投用，铁金、浩南公路建成通车，回龙湾大街等一批城市基础设施工程高效推进，城市整体面貌明显改观。

——稳步推进各项改革，行政效能和市场活力得到有效激发。完成了卫生、人口计生和公共资源交易中心等机构职能整合。理顺了城管、住建、规划等部门职责关系。组建了市公用事业投资集团。成立了中国林业产权交易所伊春交易中心。推进森工企业内部改革，探索建立了一批苗木生产、园林绿化、物业管理等实体化公司。创新农业经营主体，开展了土地流转和确权试点。规范了财税奖励政策和转移支付分配机制。取消、暂停和下放行政审批119项，清理比例达37%。

——务实开展招商引资和对外交流，多点推进对俄经贸合作。参加了央企对接、民企龙江行、中俄博览会、世界杰出华商大会等经贸活动，专程赴汉能、首创等20余家知名企业进行“一对一、点对点”对接，全年招商引资到位资金124亿元。完成了对俄跨境经济合作区境内园区规划设计，伊春·绥芬河木材储备加工物流园区有序推进。全年外贸进出口总额实现2.76亿美元，增长30.2%，位居全省第二位。

——倾力保障基本民生，人民收入水平和生活质量持续提高。全年地方财政用于民生支出65.4亿元，占比达到65%。全年城镇新增就业3.9万人。政企合一区局公务员过渡等历史难题取得突破性进展。城镇居民人均可支配收入和农民人均纯收入分别增长10%和13%。高考升学率实现“九连升”。38所公办幼儿园全部建成投用。开工建设保障性住房1.15万套、74.4万平方米。医疗卫生保障水平全面提升，安全生产、社会治理形势总体平稳，蝉联“中国幸福城市”殊荣。经过努力，承诺的10件民生实事全面完成！

此外，国防动员、民族宗教、外事侨务、妇女儿童等各项事业都取得了新进展。

各位代表，回顾过去的一年，在全国“三期叠加”、宏观经济下行压力不断加大的背景下，我们能取得这样的成绩实属不易，这是市委正确领导的结果，是全市广大干部群众团结拼搏的结果。在此，我代表市政府，向全市人民和社会各界致以崇高的敬意和衷心的感谢！

在看到成绩的同时，更要看到当前伊春转型攻坚的任务十分艰巨。主要是：森林的全面停伐、产能过剩企业的衰退、结构调整的阵痛，以及国有林区改革的深化等派生出的一系列矛盾和困难将进一步显现。伊春进入历史还债高峰期，需要较大资金去偿贷，发展的支撑力和民生保障力不足。面对转型发展的新形势，个别领导干部思想转弯慢，存在本领恐慌，一些公务人员担当意识不强，工作不在状态，缺少激情，甚至一定程度地出现懒政怠政现象。对此，我们要有清醒认识，尽最大努力应对和解决！

二、2015年工作安排

根据中央和省市委对经济形势总体判断和发展定位，今年市政府工作的指导思想是：以党的十八大，十八届三中、四中全会精神为指引，以“四个全面”战略布局为统领，以转型发展为总目标，积极主动适应经济发展新常态，认真贯彻落实中央和省市委决策部署，坚持稳中求进总基调，把转方式、调结构、攒后劲放在更加重要位置，着力抓好产业升级、生态建设、深化改革、改善民生、建设法治政府五项任务，促进全市经济平稳健康发展和社会和谐稳定。

综合各方面因素，今年全市经济社会发展的预期目标是：地区生产总值增长5.5%左右；固定资产投资增长5%；规模以上工业增加值增长5%；公共预算收入增长3.3%；城镇居民人均可支配收入增长9%，农民人均纯收入增长10%；单位GDP综合能耗下降2.1%，主要污染物排放削减率完成省下达指标。

今年，着力做好以下七个方面工作：

(一)持续推进产业结构调整。准确把握产业发展的阶段性特征，牢固树立“绿色化”发展理念，着力提升“3+X”产业的层次、质量和效益。

大力发展森林生态旅游业。全面实施《伊春旅游发展总体规划》，重点围绕“一纵两环”整合优质旅游资源，开发设计黄金线路，串联旅游精品。北部以汤旺河林海奇石国家5A级景区为核心，加快推进森林公园、湿地公园、自然保护区等景区整合升级，组团推出主打产品。中南部以铁力、桃山为核心，以伊春区为节点，整合温泉、滑雪、水上娱乐等项目和元素，统筹设计开发“大环线”，打造四季旅游产品；利用汇源绿谷、西岭度假区、带岭影视城、金山小镇、美溪回龙湾等优质资源，差异化设计开发“小环线”，打造特色旅游产品。推出以地域特色为主的文化旅游产品，做活夜场。加强与各大旅行社合作，活化营销方式。推进旅游服务体系建设，支持汇源集团建设游客到访中心，支持友好区引进战略投资者，打造旅游集散地。探索国有景区托管经营模式，全面提高旅游竞争力。

大力发展森林食品药品业。重点围绕延伸“红蓝黑黄+林药+林畜”六条主产业链，抓龙头、建基地、上项目。全力支持汇源绿谷项目群建设，加快推进中盟、黑尊、永旺等产业化龙头企业投产达效。抓好乌伊岭野生榛子林改培和友好、五营等红松嫁接果林基地建设。引导北药种植基地对接葵花、格润等企业需求，扩大返魂草、刺五加等种植规模。加速推进“伊春寒地森林猪，全程可追溯”项目基地建设，年内存栏达到20万头。积极建立生猪屠宰及冷链物流体系，努力将森林猪品牌打入上海等发达地区市场。抓好铁力现代农业示范区、嘉荫生态农业示范区建设，推动其它区局因地制宜发展特色种养业。

加强耕地保护，扎实推进农村土地经营权流转，支持农民和林业务农职工发展家庭农场、专业合作社等新型经营主体。

大力发展绿色矿业。重点在稳增长与防风险中求平衡，实施“强钼、兴玉、拓金、保钢”战略。加强与中铁资源集团等战略投资者合作，加快延伸钼产业链。做大玉街、玉石(玛瑙)批发市场，在更大范围叫响“中国桃山玉”品牌。支持中国黄金集团综合利用乌拉嘎尾矿资源，加快引进黄金制品加工生产线。鼓励并推动西钢、北方水泥等企业实现新的重组。抓住省启动地质勘查三年专项行动的有利时机，优化地质勘查环境，积极开展探矿权招商，力争地质找矿实现新突破。全力完成重点产业项目建设任务，支持铁力、翠峦等园区做大平台，进一步提升吸引和带动作用，铁力园区年内晋升为省级经济开发区。

在继续巩固壮大家具、木制工艺品、新能源等产业的同时，把提升第三产业层次和水平作为优化“X”产业的重点，大力发展现代服务业。养老养生产业，加快推进全总回龙湾职工休养中心项目建设，鼓励社会力量兴办养老机构，支持战略投资者联合医疗机构建设医养健康循环养生基地。加快发展金融业，支持国有商业银行创新金融产品，鼓励龙江银行、哈尔滨银行拓展业务，积极引进新的担保公司、投资公司和投资银行。扩大“菌联融”试点，拓宽农业融资渠道。支持兴安新能源、越橘庄园、忠芝等企业在“新三板”上市。积极发展电子商务和商贸物流业，成立伊春市电子商务协会，推进电商平台建设，引导企业和经营者主动对接东北绿色食品产业联盟、“淘宝·特色中国·伊春馆”、“1号店·特产中国·伊春县域馆”等平台和渠道，构建线上线下立体营销网络。加快建设汇源、铁力松涛物流贸易中心，谋划建设大型现代化收储冷藏库。强化商标战略，促进品牌经济发展。推进质量伊春、诚信伊春建设。利用汇源国际会展中心和现有体育、文化场馆，创新运营机制，加快发展会展、体育和文化时尚产业。

(二)加快主体生态功能区建设。对照国家《关于加快推进生态文明建设的意见》，进一步明确生态文明先行示范区建设的总体要求、目标愿景、重点任务和制度体系，系统化、累积式推进生态建设。

强化保护体系建设。全面开展林地保护专项行动，严守生态红线，严厉打击盗伐毁林、非法侵占林地湿地等行为。统筹推进防火道路、水源地建设，大力提高队伍的专业化、特种化和空中作业能力，整体提升森防现代化水平。充分发挥白头鹤保护联合会作用，进一步扩大野生动物保护范围。抓好伊春国家公园建设方案编制完善、区域生态移民等相关工作，适时启动试点。

狠抓环境污染综合防治。严守环境质量底线，严格落实节能减排主体责任，加强对重点流域内污水排放企业的监管，保证饮用水源地安全。严格控制能源消费、碳排放和主要污染物排放总量。加强与首创集团合作，加快推进双丰、新青、金山屯生物质热电联产项目建设。新建续建带岭、桃山等污水处理和乌伊岭、朗乡等生活垃圾处理工程，确保环境质量越来越好。

提升森林经营水平。系统化推进种苗繁育、造林更新、抚育设计。加强红松等珍贵树种良种基地建设，支持苗圃资产优化配置和重组，力争苗木产能达到1亿株。加大对疏林地、矿山区、采伐迹地的造林补植力度，提高水曲柳、黄菠萝、胡桃楸等珍贵用材树种造林比例，鼓励营造胡枝子等特色经济林，全年更新造林35万亩以上。加强中幼龄林抚育，全年抚育200万亩以上。

(三)全面推进关键环节改革。充分发挥改革的牵动作用，优先安排有利于稳增长、调结构、惠民生的事项，将改革的新红利转化为发展新动能。

推进林业综合改革。认真贯彻落实《国有林区改革指导意见》、《国有林场改革方案》，支持新青局开展“政企分开、管办分离”改革试点。继续推进红星局、朗乡局、铁力局营林体制、苗木生产经营机制改革。支持汤旺河局扩大碳汇林业试点，推动林业资产证券化。抓好五营局“省森工总局先行改革试点”。

推进机构改革。做好事业单位类别界定，规范机构设置。调整优化木材生产、机械、经销等机构设置，年底前整合到位。将地方林业管理职能从营林事业中剥离，建立市政府林业行政主管部门。整合市资源林政、营林等领域相关执法权，实行统一综合执法。

推进重点领域改革。稳步实施公共资源配置市场化改革，通过BOT、PPP等方式，加快推动民营资本进入公用事业领域，合理处置非经营性国有资产。探索“南四局”与铁力市共建共享机制，优化配置城镇基础设施、教育医疗等公共资源。继续细化预算编制，实现全口径预算管理。推动文艺院团加速向市场化经营转变。完成公车改革。

(四)扩大以对俄交流合作为重点的对外开放。抓住 “中蒙俄经济走廊”黑龙江陆海丝绸之路经济带建设机遇，增强机遇意识和战略意识，实现“北开”与“南联”互动发展。

提升沿边开发开放层级。加强对俄远东开发政策的研究，主动对接开放领域，整合全面停伐后各林业局闲置生产要素和骨干力量，筹建对俄开发集团，扩大林农合作，参与重大基础设施和产业项目建设。积极推进对俄跨境经济合作区、伊春·绥芬河木材储备加工物流园区建设。争取嘉荫口岸尽快恢复通关。超前做好孙吴至乌伊岭、汤旺河至富锦以及嘉荫口岸连接线等沿边铁路建设的准备工作。

务实精准招商。继续跟进对接联想、汉能、复星、宝能等战略投资者，促进意向性项目早日签约落地。面向发达地区、产业溢出地区，紧盯央企、大型民企尤其是500强企业，有针对性地设计和谋划一批重点招商项目。建立招商引资统筹合作机制，促进招商项目在各区局间合理布局。深入对接“五大规划”等政策，找准接口和切口，争取得到最大限度的支持。

(五)推动城市建设提档升级。秉持生态化、自然化理念，着力拓展城市功能，提升城市品位，加速建设美丽伊春。

统筹推进城市建设。按照中心城区功能定位，加快“四区连片”进程。支持铁力、嘉荫建设现代化生态城市。推动汤旺河、新青、金山屯、南岔、带岭、桃山等节点城镇提档升级，打造区域商业物流中心和旅游集散地。

完善城市设施功能。加快推进伊翠铁路改线工程。续建回龙湾大街。抓好伊嘉、西岭至鹿鸣等公路建设前期工作。开工建设友谊至翠宏山110千伏线路工程。加快建设黑龙江(嘉荫段)干流防洪工程。启动实施新青抗美河、援朝河、乌马河、梅花河等区段10个重点水利项目。加快推进华能热电联产项目建设，力争年内投产。加快棚改配套设施建设，盘活存量房源。

加强城市科学化管理。全面落实《伊春市城市综合管理规定》。出台《楼房外立面保温改造管理细则》。以实施《黑龙江省物业管理条例》为牵动，创新物业管理经营模式。推进智慧伊春地理信息公共服务平台建设。巩固国家卫生城创建成果，确保顺利通过国家复审。启动创建文明城市和美丽乡村、场所建设三年行动。

(六)强化科技和人才支撑。坚持把创新作为转型跨越的不竭动力，把人才作为引领发展的第一资源，促进经济发展模式由要素推动向创新驱动转变。

加强科技创新能力建设。认真落实“省千户科技型企业三年行动计划”，新增省级工程技术研究中心2户。深化与东林、东农等高校的研发合作，提升林科院、农研中心技术研发和推广能力。加快双丰农林科研所、友好省级农业科技园区、翠峦省级工业园区科技企业孵化器建设。组织实施市以上重点科技项目25项。

完善人才集聚体制机制。全面落实省《关于建立集聚人才体制机制激励人才创新创业若干政策的意见》。强化编制动态统筹管理，优先向教育、医疗、科技及重大项目建设等领域倾斜配置。采取带薪兼职、项目研发等方式柔性引才引智。

(七)扎实做好民生工作。对接落实国家各项惠民利民政策，加大财政投入力度，确保基本民生实现新的改善。

优先抓好创业就业。认真落实国家和省支持创新创业的各项政策，制定出台《鼓励森工企业机关干部带头创业实施办法》，激活林业生产要素，扩大增收渠道。实施中小企业成长工程。鼓励支持“创客”自由创业，推动“大众创业、万众创新”。

提升社会事业普惠性。推进义务教育阶段学校标准化建设。支持市一中、友好三中加快打造全省名校。支持林业中心医院、省林业二院与哈医大等外埠协作医院合作，提升诊疗水平。建设老年病专科医院，引入社会资本进行特许经营。开展“全民健身年”活动，办好全民健康运动大会。

做好社会稳定工作。完善综合管理服务功能，放大社区扁平化管理效应。准确区分涉法涉诉信访事项，畅通群众诉求渠道。严格执行新《安全生产法》，全面落实安全生产责任制，坚决防范和遏制重特大事故。构建法治化、网格化、立体化社会治安综合治理体系，提升平安伊春建设水平。

集中力量办好15件民生实事：

1、加快推进厂办大集体改革，妥善解决历史遗留的10余万集体知青工接续社会保险问题，让更多的林区建设者老有所依、老有所养。

2、采取政府为城镇参保居民购买商业保险的方式，对大额住院医疗费用进行二次核销，最高额度4万元。

3、将全市城市低保标准由月人均376元提高到440元；农村低保标准由年人均2181元提高到2700元。

4、加快推进政企合一区局公务员登记，逐步提高林业职工工资待遇。

5、开工建设中心城净水厂，改善饮用水质量。

6、改造升级华能热电配套供热管网，提高供热质量。

7、开工建设保障性住房2.2万套；继续实施老旧小区改造工程；对100万平方米旧楼房进行节能保温改造。

8、科学调整公交线路，优化站点布局，延长伊春至乌马河、翠峦、友好三条主干线路营运时间，最大程度地方便群众出行，促进中心城区繁荣。

9、实施“菜篮子”工程，新增设施果蔬基地1000亩。

10、免收中职学校农村户籍和城市贫困学生学费；对20%普通高中学生给予助学补助；一次性资助考入本科二表以上院校贫困学生1000名。

11、筹集资金6800万元，对突发性困难家庭给予应急性、过渡性救助。

12、新增养老床位1000张以上；建设10个样板型社区养老服务站和日间照料中心。

13、新建改建达标型、标准型和示范型社区综合服务中心7个，提升对城区居民的服务水平。

14、为一线环卫工人增加冬季御寒补贴和临时休息场所；一线环卫工人凭作业服和胸卡免费乘坐公交车。

15、从今年起，用三年时间，在中心城区规划建设儿童启智游憩场所，为孩子们打造一个愉悦身心的好去处。

三、全面加强法治政府建设

在新常态下加快推动伊春转型发展，要求各级政府必须用法治方式和优良作风全面提高行政效能。

(一)严格依法行政。保持简政放权力度不减，坚决落实国家和省精简的行政审批事项，全面取消自行设立的审批权限。着力优化审批流程，提高行政资源中心审批事项集中度，确保7月底前网上政务服务中心投入运行。按照时限要求，加快建立“三个清单”。切实优化发展环境，保护企业和投资者合法权益。着力完善依法决策机制，严格执行公众参与、专家论证、风险评估、合法性审查等法定决策程序。建立法律顾问制度，年内完成市区两级政府法律顾问委员会的组建工作。加强执法制度建设，细化量化行政裁量标准，加快推进综合执法。主动接受人大和政协监督，加强审计监察，充分发挥“向人民报告，请人民监督”等公众监督平台作用。全面推进政务公开，在重点公开“三重一大”、财政预算等信息的基础上，将重大项目的招投标过程、进展程度和验收情况进行定期公示。

(二)强化廉政建设。严守党的政治纪律和政治规矩，全面落实党风廉政建设主体责任，着力完善重点部门和关键岗位廉政风险防控措施，强化流程控制，规避寻租空间，从严查处违法违纪案件。厉行勤俭节约，大力压缩“三公经费”。严格执行各项廉政规定，深化群众路线教育实践活动整改落实，驰而不息地整治“四风”。扎实开展“三严三实”专题教育，抓好县处级以上领导干部这个“关键少数”，为全市干部群众树立良好形象。

(三)狠抓工作落实。各级政府和组成部门都要担当尽责，始终保持良好的精神状态，以奋发有为的士气去推动工作。要力主务实，不断提高抓落实的能力；要力戒浮躁，不搞虚功、不干“花活儿”，实实在在为林区人民干点事情。要提高工作效率，一以贯之抓好政府绩效管理工程，对确定的任务目标要雷厉风行、务实高效地推进，确保中央、省委和市委重大决策落地生根。

各位代表，今年全市各项工作任务艰巨而繁重，让我们紧紧团结在以习近平同志为总书记的党中央周围，在省委、省政府和市委的坚强领导下，充分发挥广大群众的积极性、创造性，统一思想，振奋精神，励精图治，奋发有为，以功成不必在我的姿态，以“踏石留印，抓铁有痕”的精神，狠抓各项工作的落实，全力推动伊春转型发展，为加速实现小康社会目标而不懈奋斗！
